# The Four Horsemen of the Apocalypse: Cancer, Depression, Vitamin D Deficiency, and Obesity: An Observational Study

**DOI:** 10.1155/2023/9652491

**Published:** 2023-01-17

**Authors:** Savas Tuna, Mehmet Akif Aydin, Muhammet Fatih Aydin

**Affiliations:** ^1^Department of Medical Oncology, Bahcelievler Medical Park Hospital, Istanbul, Turkey; ^2^Department of General Surgery, Altinbas University Bahcelievler Medical Park Hospital, Istanbul, Turkey; ^3^Department of Gastroenterology, Altinbas University Bahcelievler Medical Park Hospital, Istanbul, Turkey

## Abstract

**Objective:**

Studies aiming at illuminating the complex relationships between cancer, obesity, 25-hydroxy vitamin D (25-OHD) deficiency, and body fat percentage are ongoing. The objective of this study was to investigate the relationships between 25-OHD deficiency, visceral fat tissue, and the Beck Depression Inventory (BDI) in cancer patients.

**Methods:**

This study was conducted from 2013 to 2022. Patients' demographic data, such as age, sex, and body mass index (BMI), and laboratory parameters, including prealbumin, albumin, calcium, phosphorus, parathyroid hormone, 25-OHD, magnesium, hemoglobin, fat percentage, and C-reactive protein, were recorded. The Beck Depression Inventory was used to determine depression levels.

**Results:**

A total of 223 colon cancer patients aged 19–84 undergoing chemotherapy at our clinic were included in this prospective study. The male patients' mean BMI was 22.91 ± 3.74 kg/m^2^, whereas that of the female patients was 26.17 ± 3.75 kg/m^2^. The difference was statistically significant (*p* < 0.001). The mean total Beck Depression Inventory score was 13 ± 9. In this patient population, 105 (47.09%) patients had minimal depression, 69 (30.94%) had mild depression, 35 (15.70%) had moderate depression, and 14 (6.28%) had severe depression. The Beck Depression Inventory score was negatively and strongly associated with BMI and moderately and negatively associated with albumin levels.

**Conclusion:**

This study reveals a significant correlation between 25-OHD levels and the Beck Depression Inventory scores among cancer patients. We believe that 25-OHD levels may be used to determine the presence of depressive symptoms in cancer patients. However, further comprehensive multicentre studies are needed to draw more definitive conclusions.

## 1. Introduction

In recent years, the increasing prevalence of obesity has been linked to an increased incidence of cancer and associated mortality [1]. Although the association between obesity and cancer has been well established [2], the underlying mechanism has yet to be elucidated. Vitamin 25-hydroxy D (25-OHD) has been suggested to be involved in this pathway; however, the nature of this link is not fully understood [3]. The immune system and 25-OHD receptor have been suggested to mediate the link between 25-OHD deficiency and cancer, which may also be associated with obesity [4, 5]. Studies have reported that the aggressiveness of cancer is low in summer, when 25-OHD production is higher due to increased exposure to the sun, and that polymorphisms of genes encoding proteins involved in the signalling pathway of 25-OHD affect the risk of developing cancer [6]. Numerous *in vitro* studies have shown that exposure of tumour cells to high concentrations of 25-OHD compounds inhibits their proliferation [6, 7]. However, cancer also affects 25-OHD levels, and there is limited evidence that 25-OHD supplementation has an inhibitory effect on cancer cells. Overall, the extant evidence is not sufficient to draw definitive conclusions, and further studies with long-term follow-ups are needed to clarify these relationships.

Previous studies have associated 25-OHD deficiency with an increased risk of developing various cancers, obesity, type 2 diabetes mellitus, and autoimmune diseases [8–10]. Serum 25-OHD, which is the main circulating form of vitamin D, inversely correlates with body weight, body mass index (BMI), fat mass, and thus with obesity. Serum 25-OHD levels are approximately 20% lower in obese people than in nonobese individuals [11]. Therefore, the recommended 25-OHD doses are higher for obese individuals than for nonobese individuals [12]. Whether low serum 25-OHD is a cause or consequence of obesity is still the subject of debate. Parathyroid hormone (PTH) is widely used as an indicator of 25-OHD deficiency. PTH levels are higher in obese people. However, the relationship between serum calcium and PTH is left-shifted in obesity, making the interpretation of the clinical significance of high PTH levels challenging [13].

The relationship between cancer and depression is well known. The side effects of treatments, including radiotherapy and chemotherapy, and uncertainty about the future make these patients prone to depression. Moreover, evidence suggests a link between 25-OHD deficiency or insufficiency and depression. Cerebral structures such as the thalamus, amygdala, cerebellum, and hippocampus express 1*α*-hydroxylase enzymes that can metabolize 25-OHD to 1,25(OH)2D3, suggesting that 25-OHD may play an autocrine or paracrine role in the brain [14]. Consequently, 25-OHD may play a key role in the pathophysiology of depression. Moreover, 25-OHD may modulate the association between depression and inflammatory responses through its effects on the immune system [15]. Studies aiming to illuminate the complex relationships between cancer, obesity, 25-OHD deficiency, and body fat percentage are ongoing. To contribute to this effort, this study was aimed at investigating the relationships between 25-OHD deficiency, visceral fat tissue, and the Beck Depression Inventory (BDI) in cancer patients.

## 2. Material and Methods

This observational study was conducted at our hospital from 2013 to 2022 after receiving ethics approval from the institutional ethics committee (Decision No. 3/3022.K-16). All patients were informed about the objectives and procedures of the study and provided verbal and written informed consent. The study was conducted in accordance with the Declaration of Helsinki.

A total of 223 patients diagnosed with colon cancer and treated at our oncology clinic were included in the study. Patients unable to understand the content of the BDI or communicate adequately, patients with cognitive disorders, and patients younger than 18 years old were excluded from the study. All evaluations were made before the initiation of chemotherapy to avoid its potential negative impacts on the patients' moods and laboratory values.

The patients' demographic data, such as age, sex, and BMI, and laboratory parameters, including prealbumin, albumin, calcium, phosphorus, PTH, 25-OHD, magnesium, hemoglobin, fat percentage, and C-reactive protein, were recorded. BMI was calculated by dividing body weight in kilograms by the square of height in meters. Total and visceral fat masses were measured using a bioimpedance analyser (Tanita SC-330; Tanita Corporation, IL, USA). BDI was applied to assess depression levels.

### 2.1. Biochemical Analysis

For biochemical analyses, blood samples were collected into tubes containing 3.8% sodium citrate or K3 EDTA as an anticoagulant and serum separator. To obtain plasma and serum supernatants, the samples were centrifuged at 3,000 rpm for 10 min. The plasma and serum samples were kept at −80°C until analysis. Serum 25-OHD levels were determined using high-performance liquid chromatography as described by Galior et al. [16]. Nutritional status was determined by serum prealbumin concentrations.

### 2.2. Beck's Depression Inventory

All patients completed the self-reported BDI, which evaluates the intensity of depressive symptoms and attitudes [17]. Each item is scored on a 4-point Likert scale from 0 to 3. The total score ranges from 0 to 63. A score of 0–9 indicates minimal depression, a score of 10–18 indicates mild depression, a score of 19–29 indicates moderate depression, and a score of 30–63 indicates severe depression.

### 2.3. Statistical Analysis

The data were analysed using NCSS 10 (2015; NCSS, LLC, Kaysville, UT, USA) statistical software. Data normality was examined using the Kolmogorov–Smirnov test, histograms, Q-Q plots, and box plots. Continuous variables were expressed as means and standard deviations, medians, and ranges. Categorical variables were expressed as frequencies and percentages. The correlations between the BDI scores and the other variables were evaluated using Spearman's rank correlation coefficient. The BDI scores were compared between the sexes using the Mann–Whitney *U* test. Values of *p* < 0.05 were considered statistically significant.

## 3. Results

The patients' mean age was 54.60 ± 12.36 years (range: 19–84 years). Among them, 104 (46.64%) were male, and 119 (53.36%) were female. The mean BMI was 24.65 ± 3.74 kg/m^2^. The male patients' mean BMI was 22.91 ± 3.74 kg/m^2^, whereas that of the female patients was 26.17 ± 3.75 kg/m^2^. The difference was statistically significant (*p* < 0.001). The most common comorbidity was hypertension (28.70%), followed by diabetes mellitus (15.70%) and gastritis (6.28%). The patients' laboratory parameters are shown in Table 1.

The mean total BDI score was 13 ± 9. In this patient population, 105 (47.09%) patients had minimal depression, 69 (30.94%) had mild depression, 35 (15.70%) had moderate depression, and 14 (6.28%) had severe depression. The male patients' median BDI score (13, range: 2–44) was significantly higher than that of the female patients (9, range: 1–39; *p* = 0.03).

**Figure 1:**
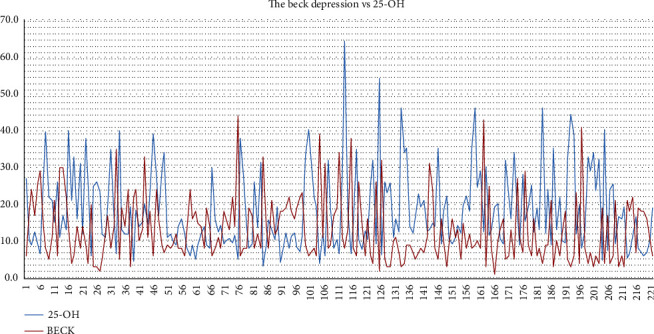
Beck's Depression Inventory vs. 25-OHD. The *X* axis shows the number of patients.

The correlations between the BDI score and the other parameters are presented in Table 2. The correlation between the BDI score and 25-OHD is shown in Figure 1. The distribution of nutritional parameter values according to depression groups is shown in Table 3.

## 4. Discussion

It is of paramount importance to monitor cancer patients' nutritional and depression status to help them adhere to treatment and improve their survival. Maintaining laboratory parameters such as albumin, hemoglobin, and 25-OHD near normal levels has positive effects on clinical outcomes.

Vitamin 25-OHD plays an important role in many biological mechanisms, and its deficiency can have numerous adverse consequences. Recent studies have focused on the relationship between 25-OHD deficiency and cancer [18]. Several meta-analyses have shown that low serum 25-OHD levels are associated with colorectal cancer [19]. In line with these findings, the mean 25-OHD concentration in this study was 18.3 ng/mL (normal range: 25–80 ng/mL). Conversely, Xu et al. found a positive correlation between serum 25-OHD levels and the risk of developing prostate cancer [20].

The effect of 25-OHD supplementation on cancer outcomes is still debated. In a study involving 25,871 participants, Manson et al. found that 25-OHD supplementation did not result in a lower incidence of invasive cancers than a placebo [21]. In contrast, Jacot et al. reported that tailored high-dose oral 25-OHD supplementation could normalize serum 25-OHD levels in women with early-stage breast cancer [22]. A meta-analysis of randomized controlled studies (RCTs) found that 25-OHD supplementation resulted in a significant reduction in total cancer mortality but not in a decrease in the total incidence of cancers [23]. Although numerous studies have investigated the relationship between 25-OHD status and the incidence of colorectal adenoma and carcinoma and associated mortality [24], a clear consensus on the effects of 25-OHD deficiency and supplementation on cancer outcomes is still lacking.

Psychometric measures are important for diagnosing and treating depression in cancer patients. BDI is an adequate tool for detecting depressive disorder in this patient population [25]. In this study, the mean total BDI score was 13 ± 9, with 47.09% of the patients having minimal depression, 30.94% having mild depression, 15.70% having moderate depression, and 6.28% having severe depression. Biracyaza et al. reported a BDI score of 16.3 [26]. Mercan et al. suggested a cut-off score of 17 for diagnosing depression among cancer patients [27]. According to Wedding et al., most BDI score alterations in cancer patients are related to somatic and not to affective symptoms and may be attributed not to depression but to the severity of the disease [28]. Conversely, other studies have found no association between BDI scores and cancer. For instance, Eskelinen and Ollonen reported that there was no specific link between breast cancer and BDI, although breast cancer patients tended to have an increased risk of developing depression [29].

In this study, the only strong correlation observed was that between BDI and BMI, with BDI scores increasing as BMI decreased. This negative correlation indicates a worsening depression status, which impacts nutrition status and decreases 25-OHD levels. In this study, the male patients had a mean BMI of 22.91 ± 3.74 kg/m^2^, whereas the female patients had a mean BMI of 26.17 ± 3.75 kg/m^2^. Accordingly, men had higher BDI scores than women. No significant correlation was found between BDI and nutritional factors (BMI, fat percentage, and visceral fat mass). The mechanisms mediating the association between malnutrition and depressive mood remain unclear and likely differ between patients.

### 4.1. Study Limitations

The most important limitations of this study are its single-centre design and the fact that it did not include a control group. An RCT involving healthy individuals might be a more appropriate design. Moreover, the role of comorbidities could have been examined in more detail. Furthermore, parameters other than BDI could be compared between the sexes. Finally, BMI could be addressed in such a way as to allow the determination of a cut-off for depressive symptoms. Despite this study's limitations and given the complexity of the issue and the lack of a clear consensus, our results can guide more comprehensive studies in the future.

## 5. Conclusion

This study reveals a significant correlation between 25-OHD levels and BDI scores among cancer patients. We believe that 25-OHD levels may be used to detect the presence of depressive symptoms in cancer patients. However, further comprehensive multicentre studies are needed to draw definitive conclusions.

## Figures and Tables

**Table 1:** Laboratory values of the patients.

Parameter	Mean	±SD	Median	Minimum	Maximum
Prealbumin (g/L)	0.213	0.071		0.095	0.430
Albumin (g/dL)	3.647	0.499		2.400	5.200
Calcium (mg/dL)	8.827	0.584		7.300	10.600
Phosphorus (mg/dL)	3.004	0.550		1.880	5.000
PTH (pg/mL)			34.700	9.500	143.200
25-OHD (ng/mL)			14.800	3.200	64.300
Magnesium (mg/dL)			2.050	1.290	4.280
Hemoglobin (g/dL)	11.470	1.365		7.400	15.900
CRP (mg/dL)			4.500	0.500	68.800
Fat %			17.200	7.000	47.200
Visceral fat mass			8.000	1.000	17.000

**Table 2:** Correlations of Beck's Depression Inventory and the other parameters.

	*r*	*p*
Age (years)	0.308	<0.001
Prealbumin (g/L)	-0.591	<0.001
Albumin (g/dL)	-0.548	<0.001
Calcium (mg/dL)	-0.304	<0.001
Phosphorus (mg/dL)	-0.166	<0.001
PTH (pg/mL)	0.463	<0.001
25-OHD (ng/mL)	-0.719	<0.001
Magnesium (mg/dL)	0.036	<0.001
Hemoglobin (g/dL)	-0.394	<0.001
BMI (kg/m^2^)	-0.204	<0.001
CRP (mg/dL)	0.248	<0.001
Fat%	-0.074	0.271
Visceral fat	-0.030	0.658

**Table 3:** Distribution of the nutritional parameters according to depression groups.

Depression severity	BMI (kg/m^2^)	Fat%	Visceral fat mass
Minimal	25.28	19.92	7.50
Mild	24.53	19.25	7.72
Moderate	23.45	19.55	7.40
Severe	23.55	17.78	7.64

## Data Availability

Data used in this study are included in the manuscript.
